# SinR is a mutational target for fine-tuning biofilm formation in laboratory-evolved strains of *Bacillus subtilis*

**DOI:** 10.1186/s12866-014-0301-8

**Published:** 2014-11-30

**Authors:** Sara A Leiman, Laura C Arboleda, Joseph S Spina, Anna L McLoon

**Affiliations:** Department of Molecular and Cellular Biology, Harvard University, Cambridge, MA 02138 USA; Biology Department, Colgate University, Hamilton, NY 13346 USA; Current address: Channing Division of Network Medicine, Brigham and Women’s Hospital and Harvard Medical School, Boston, MA 02115 USA; Current address: Department of Ecophysiology, MPI for Terrestrial Microbiology, D-35043 Marburg, Germany

**Keywords:** Adaptation, Bacteria, Biofilms, Domestication, Laboratory, Selection

## Abstract

**Background:**

Bacteria often form multicellular, organized communities known as biofilms, which protect cells from a variety of environmental stresses. During biofilm formation, bacteria secrete a species-specific matrix; in *Bacillus subtilis* biofilms, the matrix consists of protein polymers and exopolysaccharide. Many domesticated strains of *B. subtilis* have a reduced ability to form biofilms, and we conducted a two-month evolution experiment to test whether laboratory culturing provides selective pressure against biofilm formation in *B. subtilis.*

**Results:**

Bacteria grown in two-month-long batch culture rapidly diversified their biofilm-forming characteristics, exhibiting highly diverse colony morphologies on LB plates in the initial ten days of culture. Generally, this diversity decreased over time; however, multiple types of colony morphology remained in our final two-month-old populations, both under shaking and static conditions. Notably, while our final populations featured cells that produce less biofilm matrix than did the ancestor, cells overproducing biofilm matrix were present as well. We took a candidate-gene approach to identify mutations in the strains that overproduced matrix and found point mutations in the biofilm-regulatory gene *sinR*. Introducing these mutations into the ancestral strain phenocopied or partially phenocopied the evolved biofilm phenotypes.

**Conclusions:**

Our data suggest that standard laboratory culturing conditions do not rapidly select against biofilm formation. Although biofilm matrix production is often reduced in domesticated bacterial strains, we found that matrix production may still have a fitness benefit in the laboratory. We suggest that adaptive specialization of biofilm-forming species can occur through mutations that modulate biofilm formation as in *B. subtilis.*

**Electronic supplementary material:**

The online version of this article (doi:10.1186/s12866-014-0301-8) contains supplementary material, which is available to authorized users.

## Background

Many species of bacteria form multicellular communities called biofilms, in which aggregated bacterial cells are encased by an extracellular matrix that may comprise polysaccharides, proteins, and nucleic acids [1]. Despite the energetic cost of synthesizing extracellular matrix, cells in biofilms can have a fitness advantage over free-living cells [2]. Biofilms can help cells survive adverse conditions; for example, independent from genetic resistance mechanisms, cells within a biofilm are often more resistant to antibiotic treatment than are their planktonic counterparts [3,4]. Biofilms also allow bacteria to form robust communities on both biotic and abiotic surfaces, which can be ecologically beneficial in the environment but which often pose a threat in clinical and industrial settings [5,6]. Although biofilm regulatory pathways and the identities of matrix components are species-specific, the advantages of biofilm formation are widespread.

*Bacillus subtilis* is an endospore-forming bacterium that is frequently found in the soil or associated with plants, and its biofilm-forming abilities have been studied in the laboratory for over a decade [7,8]. When *B. subtilis* approaches stationary phase in biofilm-promoting media, the bacteria – initially a population of motile single cells and cell chains – aggregate and become an ordered biofilm community [9]. Within the developing biofilm, a subset of cells secrete a matrix that contains complex polysaccharides, amyloid-like fibers of the protein TasA, and the hydrophobin BslA [10-13]. Notably, the operons responsible for matrix exopolysaccharide (*epsA-O*) and matrix protein (*tapA-sipW-tasA*) are under the control of the transcriptional repressor and biofilm master regulator SinR [12,14].

In the laboratory environment, *B. subtilis* is often cultured in conditions that do not induce robust biofilm formation, such as constant aeration and the standard laboratory medium LB. Notably, supplementing LB with glycerol and additional manganese can trigger high matrix production in stationary-phase *B. subtilis* [15]. Given the diversion of resources that occurs during matrix production, we hypothesized that typical culturing conditions might select for *B. subtilis* mutants that use all of their resources for growth rather than for producing biofilm matrix, even at low levels. Laboratory strains of *B. subtilis* and of other bacterial species often form less robust biofilms than do their wild ancestors, suggesting that biofilm attenuation is common during domestication [16,17]. Although we believe that historical contingencies (irradiation and repeated transfer from laboratory to laboratory) determined which mutations actually arose in commonly-used laboratory strains of *B. subtilis* such as 168, we set out to determine whether standard laboratory conditions alone (e.g., rich liquid media, constant aeration) could have selected for the loss of biofilm formation in domesticated *B. subtilis.*

To examine biofilm formation during extended laboratory culture of *B. subtilis*, we grew multiple independent populations of a robust biofilm-forming strain of *B. subtilis*, NCIB3610 (referred to hereafter as either 3610 or the ancestor). We cultured cells for 60 days, using two different growth conditions, in LB. We regularly saved samples of each evolving population throughout the 60-day period, allowing us to monitor colony morphologies over time, and to analyze strains isolated from our final, 60-day-old populations. To our surprise, we did not uniformly re-domesticate *B. subtilis,* but rather created populations whose members form biofilms with varying levels of robustness on rich medium. Our study suggests that laboratory conditions produce both bacteria with biofilm-attenuating mutations and bacteria with biofilm-enhancing mutations. Neither class of mutation fixed in the population over 300 (shaking) or 150 (static) generations. Thus, it is unlikely that standard laboratory culturing alone led to the domestication of biofilm-forming *B. subtilis*.

## Results

### Extended culture produces coexisting cell-types with varied biofilm-forming abilities

**Figure 1:**
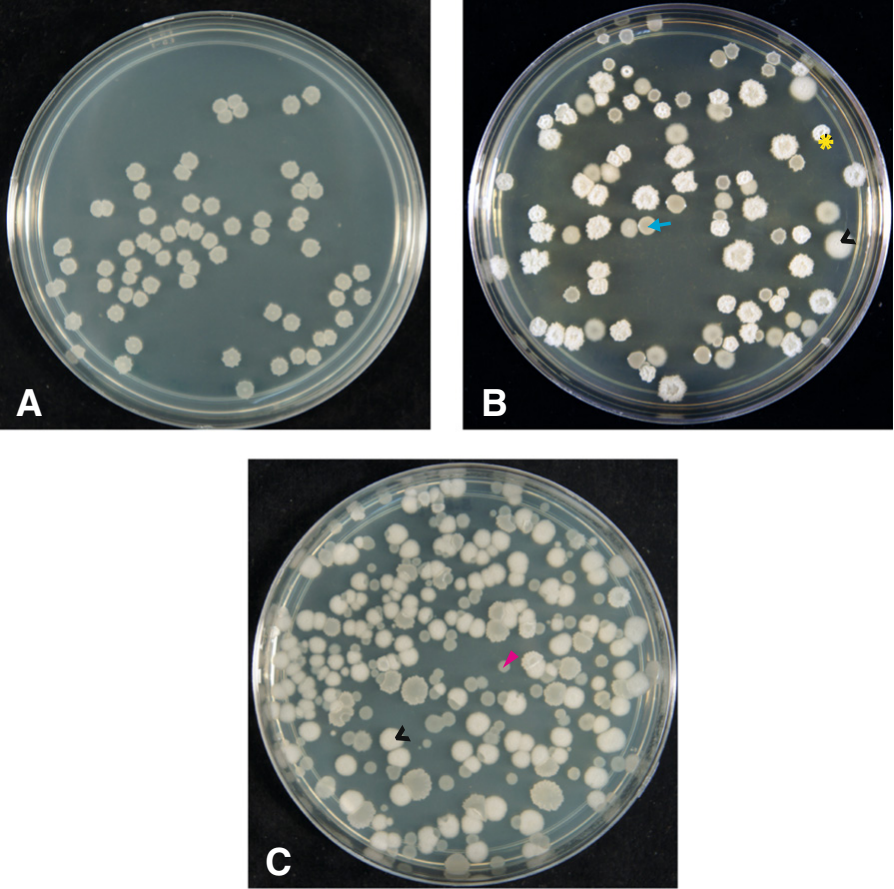
**Regardless of culture mixing strategy, over 60 days of culture,** ***B. subtilis*** **populations diversified in biofilm-forming ability, and diverse colony morphotypes were maintained.** Samples of ancestral cells **(A)** or evolved populations from the 60^th^ day of representative shaker and static cultures (**B** and **C**, respectively), were serially diluted to form individual colonies, plated on LB, and incubated for 15 hours at 37°C. Cyan arrow indicates mucoid colony, black (<) indicate fuzzy colonies, magenta arrowhead indicates smooth colonies, yellow (*) indicate wrinkled colonies. Images show standard 85 mm diameter plates.

**Figure 2:**
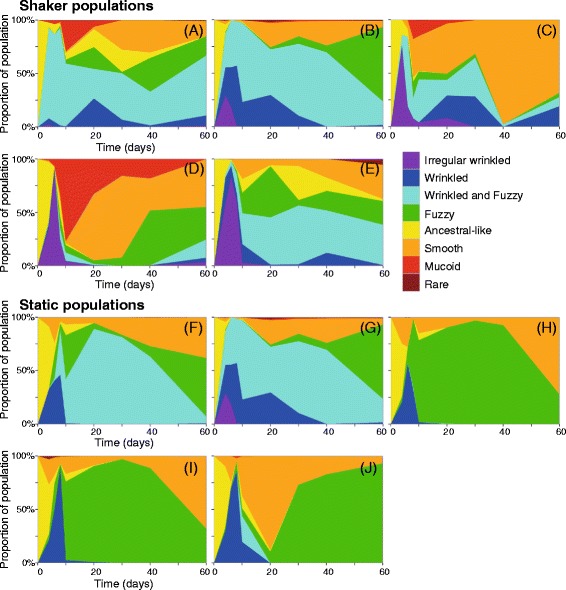
**Evolving populations show similar changes in diversity over time.** The presence and distribution of colony morphotypes over time were determined for each of the ten two-month extended cultures, using samples from days 4, 6, 8, 10, 20, 30, 40, and 60. Shaker populations 1–5 are representated in **A**-**E**, respectively, and static populations 1–5 are represented in **F-J**, respectively. The data shown represent the average of two replicates. The colors represent colony morphologies as follows: irregular wrinkled (purple), wrinkled (dark blue), wrinkled and fuzzy (FW, turquoise), fuzzy (green) ancestral-like (yellow), smooth (orange), mucoid (red), and rare morphologies (dark red).

Ten independent populations of *B. subtilis* were founded from single colonies as either shaking or static cultures of the robust biofilm-forming strain 3610 and were serially transferred daily or every second day following vigorous vortexing. The cultures were maintained for 60 days, with a calculated average of 5.645 generations per dilution, representing over 338 generations for the shaking culture, and over 169 generations for the static culture. The asynchronicity between the shaking and static cultures was chosen to compensate for the steep oxygen gradient and lower nutrient mixing of static cultures, which disadvantages cell growth. Each of the five shaking and five static populations was sampled regularly and the types and distributions of colony morphologies were examined on LB plates (Figures 1 and 2). Within the first week of culturing, cells forming non-ancestral colony types appeared in all ten cultures, and many of these diverse colony morphologies were maintained at varying proportions for the duration of the experiment (Figure 2).

We classified the colony morphologies of our evolved strains as follows, in order of most to least robust biofilm: irregular wrinkled, wrinkled, wrinkled and fuzzy (FW), fuzzy, ancestral, smooth, or mucoid (Additional file 1: Figure S1). Cells forming these diverse colony morphologies were all visible over the course of the experiment and can be seen in plated samples of the 60-day cultures (Figure 2). Cells producing wrinkled, irregular wrinkled or FW colonies rapidly appeared in the shaking cultures, nearly taking over the culture by day 6 or 8, and then decreasing in abundance over time but never completely disappearing (Figure 2A-E, purple, dark blue and turquoise). Smooth colony-forming cells, on the other hand, appeared in the shaking cultures at later days, and their frequency generally increased over time (Figure 2A, orange). Fuzzy and mucoid colony-forming cells followed less clear patterns in the shaking cultures, becoming prevalent in some cultures but not in others.

Cells forming wrinkled or irregular wrinkled colonies were generally less common in the static cultures than in shaking cultures, whereas cells forming fuzzy or FW colonies were more common (Figure 2F-J). As was the case for the shaking cultures, the prevalence of smooth colony-forming cells in the static cultures increased at later time points. Mucoid colonies were absent from the static cultures apart from static culture 5, where this morphotype never comprised more than 2% of the total population.

**Figure 3:**
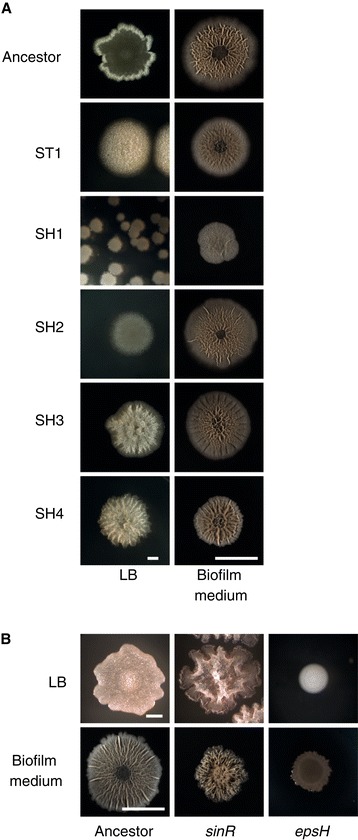
**60-day evolved strains show diverse colony architectures, with more extreme differences visible in LB colonies.** Select colony-purified evolved strains were grown from single cells on LB or from 3 μl spots on MSgg **(A)**. For comparison, LB and MSgg colony biofilms are included from deletion mutant strains Δ*sinR::spec* (DS92) and Δ*epsH::tet* (RL3852) **(B)**. Scale bars for LB colonies are 1 mm and for MSgg colonies are 1 cm.

Following two-month extended culture, we colony-purified 15 colonies from either static or shaking populations. We determined that colony morphology (as observed on LB plates) was a stable trait for the selected isolates. For this study, we then chose to limit further work to a representative sample of five isolates from the day 60 populations that represented the three most distinctive phenotypes: smooth, fuzzy, and wrinkled (Figure 3A, left images). Some strains, including SH1 (shaker 5, day 60, smooth) and SH2 (shaker 3, day 60, smooth), produced smooth, featureless colonies resembling those made by cells lacking the exopolysaccharide synthesis gene *epsH* (Figure 3B) [12,18]. Other strains, such as ST1 (static 1, day 60, fuzzy), SH3 (shaker 3, day 60, wrinkled), and SH4 (shaker 3, day 60, wrinkled), produced colonies with more surface structure than did the ancestral strain (Figure 3A). We hypothesized that the enhanced structure resulted from overproduction of extracellular matrix. Surprisingly, despite their morphological differences on LB, all but one of the isolates characterized here exhibited an ancestral-like, wrinkled colony morphology on the standard nutrient-poor, biofilm-inducing medium MSgg (Figure 3A, right images). This observation suggests that the mutations we recovered during extended culture regulate matrix production in a nutrient-dependent manner, such that matrix gene expression is attenuated or enhanced in nutrient-rich conditions (i.e. LB), but remains the same in nutrient-poor conditions (i.e. MSgg).

### Morphology robustness correlates with expression levels of matrix genes

To test our hypothesis that colony morphology on LB plates corresponds to matrix gene expression in this medium, we constructed luciferase reporters for the operons responsible for the production of matrix exopolysaccharide (*epsA-O*) and for the production of matrix proteins (*tapA-sipW-tasA*). We measured luminescence for the robust biofilm-forming strains ST1, SH3, and SH4, as well as the morphologically featureless strain SH2. Luciferase expression levels for these four evolved strains were compared to those of the ancestral strain and a mutant of the ancestral strain lacking the biofilm repressor gene *sinR,* which leads to constitutive overexpression of matrix genes [14].

**Figure 4:**
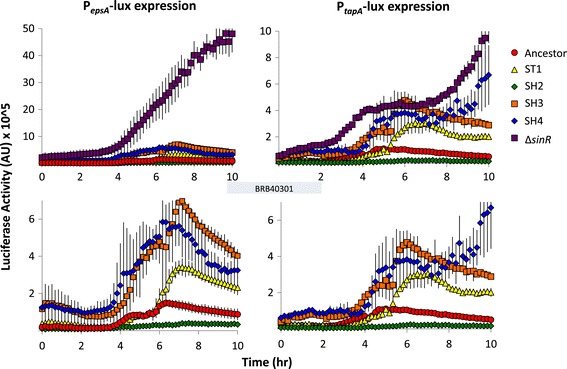
**Matrix gene expression of evolved strains corresponds to colony morphology.** Select colony-purified evolved strains, the ancestral strain, and a *sinR* null mutant of the ancestor were engineered to contain a luciferase reporter under the control of the promoter for either *epsA* or *tapA*. All strains were grown and tested in LB. Luciferase activity representative of matrix gene expression was measured every ten minutes and was normalized by culture density. The bottom panels are identical to the top panels but are re-scaled without the *sinR* null strain. Results represent the average of at least four replicates and error bars represent the standard deviation.

Matrix gene expression – as measured by luminescence - correlated with colony morphology (Figure 4). SH2 did not appear to express matrix genes over basal levels in LB, while ST1, SH3, and SH4 exhibited significantly higher matrix gene expression than that of the ancestral strain (Figure 4). Although *sinR* null mutant cells resemble the irregular wrinkled colonies observed early on in several shaking cultures (Figure 3B), none of the fuzzy or wrinkled strains obtained at the experimental endpoint of our extended culture experiment matched the extreme colony morphology or matrix gene expression levels of the *sinR* null mutant. These results further support our hypothesis that the mutations we recovered following extended culture resulted in fine-tuning of matrix expression, but caused neither constitutive expression nor, when we additionally consider the MSgg colony phenotypes, a complete absence of biofilm matrix.

### Wrinkled colony-forming evolved strains have mutations in the regulatory gene *sinR*

**Figure 5:**
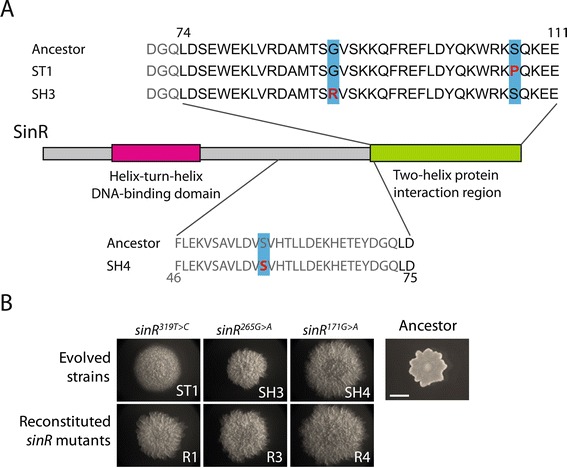
**Hyper-biofilm forming evolved strains of the fuzzy and wrinkled morphotypes have mutations in the master biofilm regulator** ***sinR.*** Schematic of the SinR protein and relevant sequence. **(A)** Red letters and blue boxes represent amino acids affected in the evolved strains. The numbers above or below the sequence indicate the amino acid number within the full-length protein. **(B)** The *sinR* mutations identified in ST1, SH3, and SH4 were transferred into the ancestral strain (from left to right, R1, R3, R4). All strains were plated on LB +1% agar, and colonies arising from single cells were photographed after 24 hours. All images were taken at the same scale. Scale bar, 250 mm.

Due to the significant role of *sinR* in biofilm development, we focused our genetic analysis of our evolved strains on the *sinR* locus. SinR tetramers act as transcriptional repressors of matrix genes during vegetative growth, whereas during stationary phase sinR monomers form a complex with either SinI or SlrR. SinI is an antirepressor and can sequester SinR, while SlrR-SinR complexes release repression of the matrix operons and instead repress genes needed for planktonic growth [14,19,20]. Sequencing the *sinR* locus revealed that strains ST1 and SH3 each carry a mutation in the protein-interaction domain of SinR (Figure 5A), which is the domain responsible for homodimerization and binding with SinI and SlrR. These mutations may modulate SinR binding, by either decreasing SinR dimerization or tetramerization, or possibly by increasing SinR-SlrR or SinI-SinR binding [21,22]. A T-to-C point mutation in ST1 changes amino acid 107 from a serine to a proline. In SH3, a G-to-A point mutation changes amino acid 89 from a glycine to an arginine. SH4 harbors a silent mutation in *sinR* at serine 57 changing the codon from TCG to TCA. Subramaniam et al. showed that serine codon bias within *sinR* serves to couple SinR translation to serine levels in the cell [23]. Interestingly, the mutation in SH4 does not fit the general model posed by Subramaniam et al., who observed that mutations changing TCA codons to TCG codons led to higher SinR production and more wrinkled colonies. Instead, we observed the opposite mutation, which nonetheless led to more wrinkled colonies. Regardless, the phenotype of SH4 could be recapitulated by introducing its silent *sinR* mutation into a wild-type background. One possible explanation is that this silent serine mutation, which is located N-terminally, could impair the stability of the *sinR* transcript (Y. Chai, personal communication).

We note that while the robust biofilm-forming strains ST1, SH3, and SH4 harbored mutations in the master biofilm regulator *sinR*, the smooth colony-forming strains SH1 and SH2 did not, nor did the smooth isolates have mutations in *sinI*, the anti-repressor of *sinR*. Given the presence of *sinR* mutations in some of our evolved strains and the fact that SinR is involved in regulating sporulation initiation in addition to biofilm formation, we recognized the possibility that sporulation was also affected in our extended cultures. Thus, we specifically induced and measured sporulation in the evolved isolates and the ancestral strain. Our results did not indicate a correlation between the presence of a mutation in *sinR* and sporulation efficiency (Additional file 2: Figure S2).

### Point mutations in *sinR* contribute to the colony morphologies of evolved strains

Although the identified *sinR* mutations seemed likely to be responsible for the observed biofilm phenotypes in the evolved strains, we wanted to rule out a coincidental role for these mutations. To verify that the *sinR* mutations in our evolved strains were sufficient to achieve enhanced biofilm robustness, we introduced these specific point mutations into the ancestral strain. Specifically, we reconstituted the *sinR* point mutations from ST1, SH3, or SH4 in the ancestor to create the strains R1 (*sinR*^319T>C^), R3 (*sinR*^265G>A^), andR4 (*sinR*^171G>A^), respectively. As demonstrated by colony phenotype on LB agar, each of the reconstituted strains exhibits significant matrix overexpression when compared to the ancestor (Figure 5B). R3 and R4 closely phenocopied SH3 and SH4, respectively, indicating that these *sinR* mutations alone may be responsible for the evolved colony morphologies. R1 partially phenocopied the fuzzy colony-producing strain ST1 (Figure 5B). R1 had more pronounced biofilm structure than did ST1, suggesting that ST1 harbors at least one other mutation that tempers the phenotype caused by the *sinR* mutation. Overall, we found that point mutations in *sinR* arose readily during extended culture of *B. subtilis*, that these mutations are sufficient for robust biofilm formation in wrinkled colony types, and that these mutations can contribute to fuzzy colony types as well.

## Discussion

We conducted a two-month long-term evolution experiment to determine how extended laboratory culture changes biofilm formation in *B. subtilis*. Although laboratory-domesticated strains of *B. subtilis* do not form robust biofilms, two months of culture of the wild ancestor resulted in the emergence of strains with a diversity of biofilm robustness, as demonstrated by colony phenotype and the expression of matrix-producing genes. While the appearance and prevalence of strain types in each evolving culture varied over time and among replicate populations, none of our culture conditions reproducibly led to uniform attenuation or loss of biofilm formation. Instead, many of the evolved strains formed more robust biofilms than the ancestral strain and these appeared early in our populations and were thereafter present in high numbers. This suggests that matrix overproduction can be neutral, if not advantageous, even in rich medium.

We attempted to test the fitness of our evolved matrix-overproducing strains in competition with the ancestral strain and with the smooth colony variants. Unfortunately, the matrix-overproducing strains form aggregates, making these experiments highly variable and impracticable to interpret (data not shown). The same issue presented in our attempts to perform growth curves using spectrophotometric or plating methods, as the aggregates of matrix-overexpressing strains did not disperse, even following vigorous vortexing. Nonetheless, with the abundance of fuzzy and/or wrinkled colony-forming cells frequently in excess of 90% of the population, we feel confident that these cells have a strong selective advantage during 60 days of culture.

The high proportion and persistence of fuzzy colony-producing cells in particular may indicate that a moderate rise in biofilm matrix production grants cells the largest fitness advantage. Perhaps these cells are able to adhere to and colonize niches, such as the wall of a tube, unavailable to cells that make lower levels of matrix. Similarly, it is plausible that fuzzy colony-forming cells have greater fitness over robustly wrinkled colony-forming cells, because the former can allocate more resources toward proliferation.

The diversity of smooth, fuzzy, and wrinkled colonies that we observed after extended culture is reminiscent of the phenotypes observed by Rainey and Travisano in spatially structured cultures of *Pseudomonas fluorescens* [24]. Their work also led to fuzzy and wrinkled colony types, and similarly, their wrinkled phenotypes were due to mutations leading to overproduction of a polymer matrix [25]. Such results suggest that hyper-production of matrix is a common adaptation that allows bacteria to utilize the ecological niche of a culture’s air-medium or vessel-air-medium interface.

Finally, some strains in our final populations exhibited reduced colony complexity and partly resembled domesticated laboratory strains, and these became more prevalent towards the end of our experiment. It is possible that over longer culturing, they might win out due to clonal interference or drift, even though non-matrix-forming strains did not increase to fixation within our populations during the observed generations. We hope to explore the genetic bases for the observed biofilm attenuation in future work. The production of matrix is energetically costly, but is clearly not without benefits, even in standard laboratory conditions.

## Conclusions

Although biofilm formation has well-accepted advantages under many “real-world” conditions, the attenuation of biofilm formation in some laboratory bacterial strains raises questions of the fitness effects of biofilm formation under laboratory conditions. Here, we demonstrate that biofilm formation can still be advantageous in the laboratory for *B. subtilis*, and that moderate changes in matrix production may allow bacteria to colonize specific niches even in seemingly homogeneous cultures. Further, we identified several specific mutations in one key matrix regulatory gene, *sinR*, which led to increased production of matrix in subsets of our evolved populations. Because biofilm formation evolves rapidly and frequently during laboratory culture, our results suggest that biofilm-deficient laboratory strains of *B. subtilis* were selected by scientists rather than by culture conditions alone.

## Methods

### Strains and culture conditions

The strains used in this study are listed in Table 1. Strains were routinely grown in LB medium (10 g/liter tryptone, 5 g/liter yeast extract, 10 g/liter NaCl), TY medium (LB supplemented with 10 mM MgSO_4_ and 100 μM MnSO_4_), MSgg medium (5 mM potassium phosphate, 100 mM morpholinepropanesulfonic acid [MOPS] pH 7, 2 mM MgCl_2_, 50 μM MnCl_2_, 50 μM FeCl_3_, 700 μM CaCl_2_,1 μM ZnCl_2_, 2 μM thiamine, 0.5% glycerol, 0.5% glutamate, 50 μg/ml threonine, tryptophan, and phenylalanine), or Difco Sporulation medium [26]. Colonies initiated from single cells were grown in LB for 15 hours at 37°C from serially diluted starter cultures. MSgg colony biofilms were inoculated with 3 μl of starter culture, allowed to dry, and incubated for 3 days at 30°C. As appropriate, antibiotics were added at the following concentrations: tetracycline (10 μg/ml), chloramphenicol (5 μg/ml), kanamycin (5 μg/ml), X-Gal (5-bromo-4-chloro-3-indolyl-β-D-galactopyranoside; 100 μg/ml), 1 μg/ml erythromycin, and 25 μg/ml lincomycin.

**Table 1:** **Bacterial strains used in this work**

**Strain name**	**Genotype**	**Source or strain construction**
NCIB3610	ancestral biofilm-forming strain	Laboratory stock
PY79	domesticated laboratory strain	Laboratory stock
168	domesticated laboratory strain	Laboratory stock
DS92	Δ*sinR*::spec	Kearns et al. [14]
RL3852	Δ*epsH*::tet	Kearns et al. [14]
R1	*sinR* ^319T>C^ in NCIB3610	This study
R3	*sinR* ^265G>A^ in NCIB3610	This study
R4	*sinR* ^171G>A^ in NCIB3610	This study
ST1	evolved strain isolated from static culture 1 on day 60	This study
SH1	evolved strain isolated from shaking culture 5 on day 60	This study
SH2	evolved strain isolated from shaking culture 3 on day 60	This study
SH3	evolved strain isolated from shaking culture 3 on day 60	This study
SH4	evolved strain isolated from shaking culture 3 on day 60	This study
ALM89	*sacA::P*_*epsA*_ *-lux* in NCIB3610, Cm^R^	This study
SLH20	*sacA::P*_*epsA*_ *-lux* in ST1, Cm^R^	This study
SLH21	*sacA::P*_*epsA*_ *-lux* in SH2, Cm^R^	This study
SLH22	*sacA::P*_*epsA*_ *-lux* in SH3, Cm^R^	This study
SLH23	*sacA::P*_*epsA*_ *-lux* in SH4, Cm^R^	This study
SLH24	*sacA::P*_*epsA*_ *-lux* in DS92, Cm^R^	This study
ALM91	*sacA::P*_*tapA*_ *-lux* in NCIB3610, Cm^R^	This study
SLH25	*sacA::P*_*tapA*_ *-lux* in ST1, Cm^R^	This study
SLH26	*sacA::P*_*tapA*_ *-lux* in SH2, Cm^R^	This study
SLH27	*sacA::P*_*tapA*_ *-lux* in SH3, Cm^R^	This study
SLH28	*sacA::P*_*tapA*_ *-lux* in SH4, Cm^R^	This study
SLH29	*sacA::P*_*tapA*_ *-lux* in DS92, Cm^R^	This study

### Strain construction

Promoters to the *epsA* and *tapA* operons were PCR amplified from genomic DNA using 5′-TGGCGAATTCTGTACGGCTTGCACTAAATGTAC-3′ and 5′-GTTCGTCGACATTCATAGCCTTCAGCCTTCCCG-3′ (*epsA*), and 5′-GTTCGTCGACATCTTACCTCCTGTAAAACACTG-3′ and 5′-TGGCGAATTCATAGACAAATCACACATTGTTTG-3′ (*tapA*) primers, cloned into plasmid pAH321 and transferred into domesticated *B. subtilis* strain PY79 as previously described [27,28]. Reporter constructs and marker-linked mutations or gene knockouts were moved into the appropriate strains by transduction with the Spp1 bacteriophage following previously described methods [29].

Mutated *sinR* was amplified from the evolved isolates using the following primers: 5′-CGTTGTAAAACGACGGCCAGTGAATTCGTCTTCACCTAGTCTCTGGAAC-3′ and 5′-AACAGCTATGACCATGATTACGCCAAGCTTCATTCAATAAAAGGGGAGCTTACC-3′. Mutations in sinR were markerlessly reconstructed in B. subtilis 3610 using the pMiniMAD protocol as described by Patrick and Kearns [30]. Successful double-crossover events were indicated by the absence of growth in the presence of erythromycin and lincomycin. Mutations in sinR were subsequently verified using 5′-TGGATCAAGAATGGGTTGAATTAATGGT-3′ and 5′-CAGCGCCATTAGAGAAATTGAAAGAAAG-3′.

### Extended batch culture

Five replicate 5 ml LB cultures in glass test tubes were inoculated with single colonies of NCIB3610 and incubated at 37°C in an orbital shaker (150 rpm). Every 24 hours, for 60 days, the cultures were vortexed for 30 seconds, then 100 μl of the shaking cultures were transferred to fresh 5 mL LB cultures. This transfer scheme represented 10^6^-10^8^ cells transferred, and we hoped would minimize inadvertent bottleneck events or the accidental exclusion of clumpy cell types that would be underrepresented in smaller volumes. Alternatively, five replicate 5 ml LB cultures in glass test tubes were inoculated with single colonies of NCIB3610 and incubated statically at 37°C. Every 48 hours, the cultures were vigorously vortexed for 30 seconds and 100 μl were transferred to fresh LB cultures, for 60 total days. Every other day for the first 3 weeks and every week thereafter, samples from each culture were taken and stored at −80°C in 20% glycerol. Cultures were also regularly tested for contamination by plating serial dilutions of the evolving populations.

### Quantification of colony morphologies over time

Two samples of each population from days 4, 6, 8, 10, 20, 30, 40, and 60 were grown from glycerol stocks for 3 hours at 37°C in 3 mL LB. The samples were then serially diluted and plated on LB agar plates, incubated for 15 hours at 37°C, and imaged by digital photography. All colonies on each plate were counted, with a minimum of 50 colonies and an average of 150–200 colonies counted per sample. The resulting colonies were counted and classified by morphology and the relative abundances of each morphology were averaged and graphed using the R package ggplot2 [31].

### Sequencing

*sinR* was sequenced using the following primers for amplification and sequencing 5′-TGGATCAAGAATGGGTTGAATTAATGGT-3′ and 5′-CAGCGCCATTAGAGAAATTGAAAGAAAG-3′.

### Kinetic luciferase assay

Cells were grown in LB to mid-log phase and diluted 1:100 in fresh LB. Dilutions were plated in quadruplet (250 μl each) in a 96-well polystyrene Costar plate (white with a clear bottom; Fisher Scientific, USA). The luciferase activity of each strain was measured on a BioTek Synergy 2 luminometer (BioTek, USA) with continuous slow shaking at 30°C. Luciferase luminescence was measured at a sensitivity setting of 200, and culture optical density was measured at 600 nm every 10 minutes for 24 hours. Final luciferase activity values were calculated by normalizing luciferase luminescence to culture density. Data shown represents the average of at least four biological replicates.

### Spore counts

Heat-resistant spores of the ancestral and selected evolved strains were produced, isolated and counted as previously described [26]. Spores for each strain were counted in triplicate and the entire experiment was replicated three times.

## Additional files

Additional file 1: Figure S1. Representative colonies for each class of colony morphology. Images represent individual colonies from the plates featured in Figure 2. The colony diameter is in part affected by the colony density on the plates. Colony morphologies are as follows: irregular wrinkled (A,B), wrinkled (C-E), wrinkled and fuzzy (FW) (F), fuzzy (G) ancestral-like (H), smooth (I,J), and mucoid (K,L). Scale bar, 500 mm. Additional file 2: Figure S2. Spore counts for the ancestor and select evolved strains. The indicated strains were grown in liquid DSM for 28 hours, after which heat-resistant spores were isolated and plated. The resulting colonies were counted in triplicate for each strain. The data shown represent the average of three independent experiments (normalized to the ancestor for each experiment) and the error bars represent the standard deviation.
