# Supplementary figures and images for: SinR is a mutational target for fine-tuning biofilm formation in laboratory-evolved strains of Bacillus subtilis
Source: BMC Microbiol. 2014 Nov 30;14:301. doi: 10.1186/s12866-014-0301-8 (PMC4258274; doi:10.1186/s12866-014-0301-8)

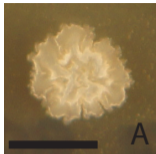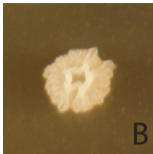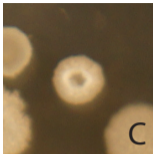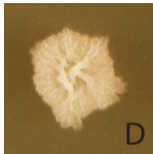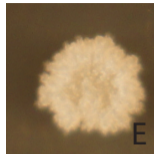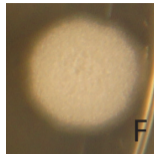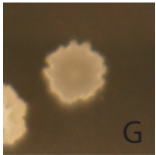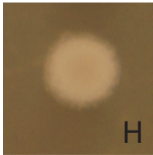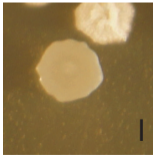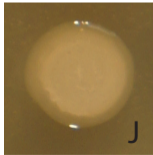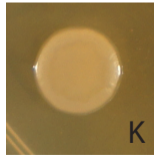

Supplement: Additional file 1: Figure S1. — Representative colonies for each class of colony morphology. Images represent individual colonies from the plates featured in Figure 2. The colony diameter is in part affected by the colony density on the plates. Colony morphologies are as follows: irregular wrinkled (A,B), wrinkled (C-E), wrinkled and fuzzy (FW) (F), fuzzy (G) ancestral-like (H), smooth (I,J), and mucoid (K,L). Scale bar, 500 mm. [file 12866_2014_301_MOESM1_ESM.pdf]

## Slide 1
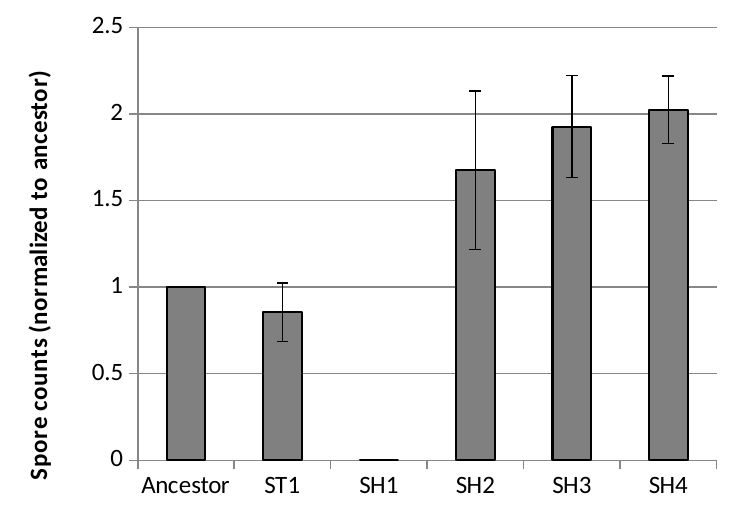

### Chart
| Category | ave |
|---|---|
| Ancestor | 1.0 |
| ST1 | 0.8551239029426948 |
| SH1 | 0.0003513485675986562 |
| SH2 | 1.6765692183910126 |
| SH3 | 1.9274164545764683 |
| SH4 | 2.025509695767555 |

Supplement: Additional file 2: Figure S2. — Spore counts for the ancestor and select evolved strains. The indicated strains were grown in liquid DSM for 28 hours, after which heat-resistant spores were isolated and plated. The resulting colonies were counted in triplicate for each strain. The data shown represent the average of three independent experiments (normalized to the ancestor for each experiment) and the error bars represent the standard deviation. [file 12866_2014_301_MOESM2_ESM.pptx]
